# Can Right Ventricular Pacing be Useful in the Assessment of Cavo-tricuspid Isthumus Block?

**Published:** 2008-11-01

**Authors:** Gennaro Miracapillo, Alessandro Costoli, Luigi Addonisio, Marco Breschi, Silva Severi

**Affiliations:** Division of Cardiology, Misericordia Hospital, Grosseto, Italy

**Keywords:** Atrial flutter, Radiofrequency ablation, Right ventricle pacing, Coronary sinus pacing

## Abstract

**Background:**

Cavo-tricuspid isthmus (CTI) block is currently assessed by coronary sinus (CS) pacing or low lateral and septal atrial pacing. Occasionally, CS catheterization through the femoral route can be difficult to perform or right atrial pacing can be problematic because of catheter instability or saturation of the atrial electrograms recorded near the catheter.

**Objectives:**

Our aim was to evaluate the feasibility of assessing cavo-tricuspid isthmus block by means of right ventricular (RV) pacing in patients with ventriculo-atrial conduction, comparing it with CS pacing.

**Methods:**

Circumannular activation was analyzed during CS and RV pacing in consecutive patients in sinus rhythm undergoing CTI ablation for typical atrial flutter. Patients without ventriculo-atrial conduction were excluded from the study. The linear lesion was created during RV pacing and split atrial signals on the ablation line were analyzed. CTI block was confirmed by analyzing local electrograms on the line of block and circumannular activation during CS and RV pacing.

**Results:**

Out of 31 patients, 20 displayed ventriculo-atrial conduction (64%) and were included in the study. Before ablation, during RV stimulation, the collision front of circumannular activation shifted counterclockwise in contrast with the pattern observed during CS pacing. After ablation, circumannular activation was similar during CS and RV pacing, showing fully descending lateral right atrium activation, even if double potentials registered on the ablation line were less widely split during RV pacing than CS pacing (111±26 ms vs 128±30 , p=0.0001).

**Conclusions:**

In patients with ventriculo-atrial conduction, tricuspid annulus activation during CS and RV pacing is similar, before and after CTI ablation. The occurrence of split atrial electrograms separated by an isoelectric interval registered on the line of block can be detected during CS or RV pacing. In patients with difficult  CS catheterization via the femoral vein, before trying the subclavian or internal jugular  route,  if retrograde ventriculo-atrial conduction is present, RV  pacing can be an easy trick to assess isthmus block.

## Introduction

The end-point of radiofrequency (RF) ablation of typical atrial flutter (AFl) is the creation of a bidirectional conduction block between the inferior vena cava (IVC) and the tricuspid annulus (TA), the so-called cavo-tricuspid isthmus (CTI) [1-4]. Validation of CTI block depends on the assessment of counter-clockwise depolarization of the TA while pacing from the septal side of the ablation line, with fully descending activation of the right lateral atrial wall, and clockwise propagation during inferior lateral atrial wall stimulation. According to some authors [5-7] the presence of a corridor of split electrograms along the ablation line is further evidence of a complete isthmus block. The coronary sinus (CS) ostium  or the low septal atrial wall are elective sites for pacing in order to assess isthmus block; because they are both close and orthogonal to CTI, the activation potentials are soon obliged to stop on one side of the ablation line and to run around the TA to depolarize the other side when CTI block occurs. Another site that could meet these requirements is the region around the AV node. Pacing can be carried out from this site indirectly, as recently suggested by Vijayaraman et al. [8], who compared right ventricular (RV) pacing with low septal atrial wall pacing in patients undergoing isthmus-dependent AFl ablation. Following a previous report of our group [9], the aim of this study was to compare CS and RV pacing in the assessment of CTI block in patients with intact ventriculo-atrial (VA) conduction, analyzing circumannular activation and double potentials on the line of block. We propose this technique when CS catheterization through the femoral vein approach is failed, before trying the subclavian or internal jugular route.

## Methods

**Figure 1:**
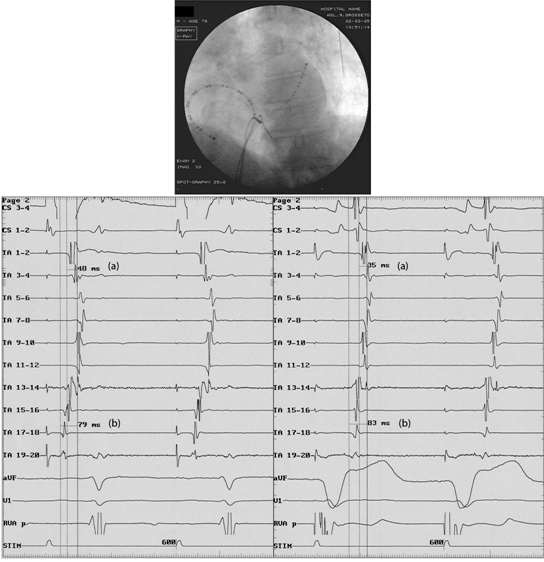
Patient 12: electrogram tracings and fluoroscopic image in the left anterior oblique 45º projection. In patients in sinus rhythm, when the line of block is not created, on pacing from CS (left side panel) and RV (right side), using the same catheter set-up (above), TA activation is similar, except for the counterclockwise shifting of the collision front (from TA 7-8 during CS pacing to TA 5-6 during RV pacing). The 'difference of circumannular activation time' was defined as the time from proximal pole to collision point (b) minus the time from distal pole to collision point (a) recorded  by the duo-decapolar catheter. These values were measured during RV and CS pacing at the same cycle length and compared. (TA 1-20: tricuspidal annulus; CS 1-4: coronary sinus; RV: right ventricle).

Consecutive patients undergoing CTI RF ablation of typical AFl were proposed for inclusion in the study. All patients gave written informed consent. Electrophysiological study was performed by using 3 catheters inserted via the right and left femoral or left subclavian veins: a quadripolar diagnostic catheter in the RV apex (Explorer ST, Boston Scientific EPT, Boston, MA, USA), a decapolar in the CS (Marinr CS, Medtronic, Minneapolis, MN, USA) and a duo-decapolar around the TA (Stablemapr, Medtronic, Minneapolis, MN, USA, or Halo XP, Biosense Webster, Diamond Bar, CA, USA). An 8 mm tip ablation catheter (Blazer XP large standard curve, Boston Scientific EPT, Boston, MA, USA) was inserted into the right atrium (RA) from the right femoral vein. In those patients who were in AFl, isthmus dependence was assessed by the entrainment technique before beginning the ablation procedure. In patients in sinus rhythm at the beginning of the study and in those in AFl whose sinus rhythm was restored during RF delivery, circumannular activation was registered during proximal CS and RV pacing. Efforts were made to pace the left atrium as nearer as possible to the CS ostium, consistently with pacing threshold and catheter stability and avoiding left ventricular pacing.   When the line of block had not yet been created, the 'difference of circumannular activation time' was defined as the time from proximal pole to collision point  minus the time from distal pole to collision point, recorded on the duo-decapolar catheter  during RV and CS pacing at the same cycle length (Figure 1). Patients with no evidence of retrograde VA conduction were excluded from the study. The presence of accessory pathways was another criterion of exclusion from the study. In the absence of isthmus block, the linear RF lesion was created during RV pacing, on observing real-time signals from the ablator only. Simultaneous blinded recordings of TA and CS signals were observed off-line once the CTI was deemed to be blocked.

Bipolar digitized endocardial atrial and ventricular electrograms and surface ECG leads were simultaneously recorded (1 KHz sampling frequency, 30-500 Hz band-pass filters), displayed on a multi-channel recorder and stored on magneto-optical disks (EP MedSystems Inc., West Berlin, New Jersey, USA). RF ablation was performed with an EPT-1000 XP generator (EP Technologies, Mountain View,  CA,  USA)  that  delivered  continuous  unmodulated  current  at  500  KHz  in  a monopolar fashion  programmed at a maximum  power output of 100 W. RF pulses were delivered through the 8 mm tip electrode catheter  in a temperature-guided mode (preset temperature of 70ºC). The preset duration of the pulse was 60 s.

**Figure 2:**
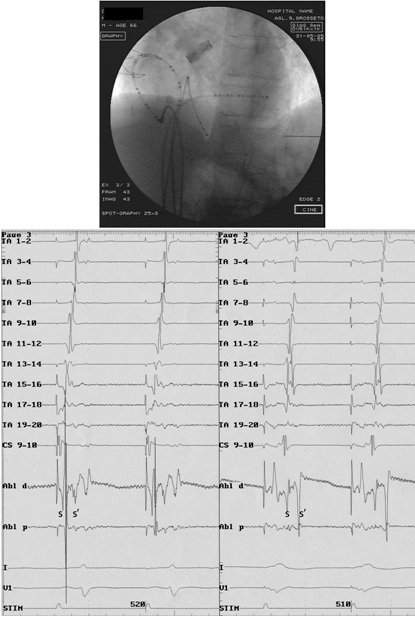
Patient 17: electrogram tracings and fluoroscopic image in the left anterior oblique 45º projection. Once the CTI has been definitely blocked, a completely descending wave front along the lateral right atrium is observed during both CS (left side panel) and RV (right side panel) pacing (TA 1-20: tricuspidal annulus; CS 9-10: coronary sinus; RV: right ventricle). Time between split atrial potentials along the ablation line after the creation of isthmus block was measured and analyzed during both CS and RV pacing (s and s').

When patients were in sinus rhythm, the procedure was performed while pacing from the RV apex at a cycle length adapted to the individual retrograde conduction properties, 600 ms if possible, at twice the diastolic threshold. The ablation catheter was initially positioned, during sinus rhythm, close to the TA where a large ventricular potential and a small atrial electrogram were recorded (A/V ratio approximately 0.1). RV pacing was then started and the catheter was progressively drawn back in a posteroseptal direction under fluoroscopic guidance, with sequential stops between each RF delivery up to the edge of the IVC. At each step, the modifications of atrial potentials related to VA conduction  were observed in order to control the progression of the ablation line. The endpoint of the procedure was the achievement of a complete clockwise isthmus block. In the first instance, this endpoint was evaluated on the basis of evidence of a sudden increase in the distance between split, reduced-amplitude atrial potentials separated by an isoelectric interval registered after the ventricular electrogram during RV pacing in the corridor between the TA and the IVC edge. The presence of sites of recording of  single atrial potentials after the ventricular electrograms, or fragmented atrial potentials without an isoelectric interval between them, suggested the presence of conduction gaps. Complete block was then validated by analyzing local electrograms registered on the line of block and circumannular activation on the duo-decapolar catheter during low lateral RA and CS pacing. This activation model was then compared with the circumannular activation registered when pacing was performed from the RV (Figure 2). The time between split atrial potentials along the ablation line after the creation of isthmus block was measured and analyzed during both CS and RV pacing. The persistence of  isthmus block was assessed 20 minutes after the last RF pulse in order to exclude the early recovery of CTI conduction. Continuous variables are reported as mean value ± standard deviation and analyzed by means of two-tailed paired Student t test. Discrete variables are reported as percentages. A p value <0.05 was considered statistically significant. Statistical analysis was made by means of the SPSS statistical software package (version 12.0, SPSS Inc., Chicago, Illinois, USA).

## Results

**Figure 3:**
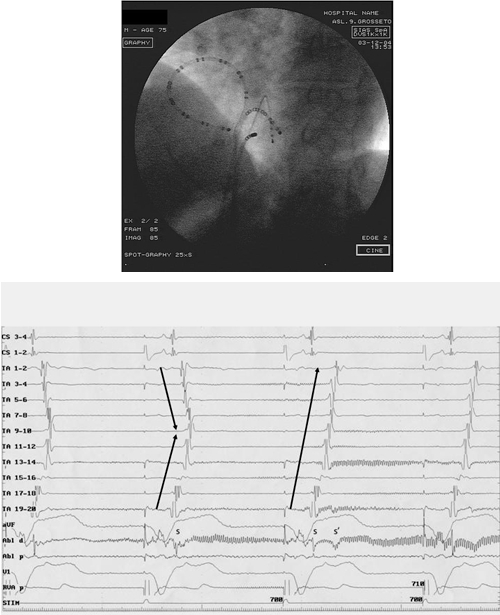
Patient 9: electrogram tracings and fluoroscopic image in the left anterior oblique 45º projection. During RV pacing, the achievement of a complete CTI block, as evidenced by the changing of the TA activation potentials (arrows) with a clear change in the polarity  of the atrial electrograms from TA1-2 to TA5-6, correlated with a sudden increase (a sort of "isthmic jump") in the distance between the two atrial components induced by retrograde VA conduction and recorded on the ablation catheter during RF delivery. (CS1-4: coronary sinus; TA1-20: tricuspidal annulus; Abl d: ablator distal poles, evidencing the splitting of the double potentials s-s', RVA: right ventricular apex).

Out of 31 consecutive patients who underwent CTI RF ablation, 20 (males 80%, mean age 72±7 years, height 171±5 cm, weight 77±12 Kg) displayed VA conduction (64%) and were included in the study. No patients evidenced accessory pathways, nor had a permanent pacemaker. At the beginning of the procedure, 11 patients were in sinus rhythm, whereas 9 had typical AFl, with a mean cycle length of 222 ± 22 ms (range 181-250). In all  patients with AFl at the beginning of the procedure, atrial potentials recorded on the CTI coincided  with the plateau between F waves on the surface electrocardiogram, and the entrainment technique showed that the CTI was the critical zone of the re-entry circuit. In all of these patients, the arrhythmia was interrupted by RF delivery, but only in one of them complete CTI block was present when sinus rhythm was restored; in all the others, further RF pulses were necessary to eliminate conduction gaps. In all patients in sinus rhythm, including those in AFl whose sinus rhythm had been restored during the ablation procedure without achieving a complete CTI block, RF pulses were delivered during RV pacing, and electrograms were monitored to detect splitting of double potentials recorded  on  the  ablation   line   after   the ventricular signals. In all patients, the achievement of a complete CTI block (subsequently verified by CS pacing) correlated with a sudden increase in the distance between the two atrial components induced by retrograde VA conduction and recorded on the ablation catheter during RF delivery (Figure 3). Thus, once complete CTI block had been achieved, a corridor of split atrial potentials was present all along the ablation line during  RV pacing, after the ventricular electrograms. Comparison of atrial potentials recorded on the ablation line during RV and CS  pacing revealed that double potentials were more widely split during CS pacing than during RV pacing (128±30 ms vs. 111±26 ms, p=0.0001). A mean of 12 RF pulses (range 4-30) were delivered. All patients were successfully ablated and no complications occurred.

Circumannular activation was analyzed before and after the achievement of CTI block in all patients but one; in this case, AFl was interrupted during RF ablation, with immediate creation of a complete CTI block and pre-block circumannular activation could not be analyzed. Before ablation,  the collision front of the circumannular activation during RV stimulation shifted counterclockwise in comparison with CS pacing, with a significant increase  in the difference  of activation time registered on the duo-decapolar catheter (59±26 ms vs 38±28, p=0.001). After ablation, the circumannular activation was very similar during CS and RV pacing, showing fully descending lateral RA activation. Following the creation of bidirectional block, according to previous findings [8] a clear change in the polarity  of the atrial electrograms lateral to the isthmus ablation line was noted during RV pacing (Figure 3). During a mean follow-up of 30 days, no patients complained of AF1 recurrences nor suffered episodes of paroxysmal atrial fibrillation.

## Discussion

CS pacing, low lateral and septal atrial pacing are validated techniques to assess  true complete isthmus block [10-14]. In those few cases  where CS catheterization is difficult or RA pacing is unstable or problematic, in agreement with previous studies[8], we confirm the utility of a little trick that  may be helpful: pacing from RV can mimic CS pacing if VA conduction is present.

### Circumannular activation analysis

**Figure 4:**
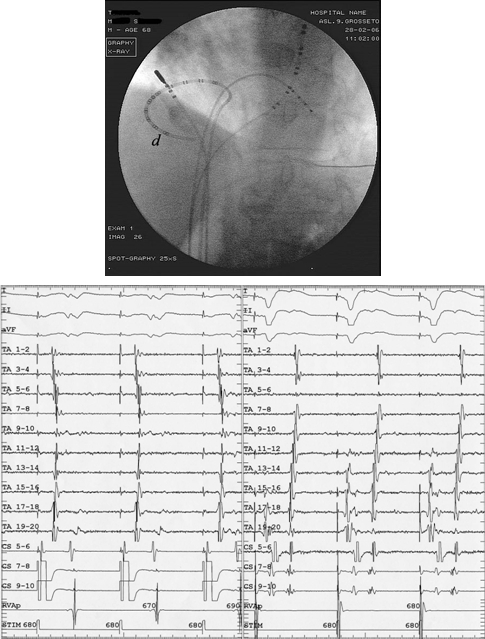
Patient 26: electrogram tracings and fluoroscopic image in the left anterior oblique 45º projection. (TA 1-20: tricuspidal annulus; CS 5-10: coronary sinus; RVA: right ventricle apex). In this case, TA activation evidences a clear shift of the collision front from TA 7-8 during CS pacing (left panel) to TA 3-4 during RV pacing (right panel). The fact that the AV node is located more anterior than the CS ostium means that, when pacing is carried out from the RV instead of the CS, TA activation shifts counterclockwise. Because pacing from the RV can make the collision front shift towards the CTI, careful mapping of the inferior lateral atrial wall is mandatory in order to avoid the mistake of regarding an isthmus as blocked when it is not. In this case, as the distal pole (d) of the TA mapping catheter was spaced 18 mm from the tip, the inferior lateral atrial wall was not completely mapped.

Analysis of circumannular activation on the basis of the potentials recorded by the duo-decapolar catheter revealed a similar configuration during RV and CS pacing in patients without CTI block before the ablation procedure. There are, however, some important differences: during CS pacing, the atrial electrograms precede the ventricular potentials, while during RV pacing they follow; when RV pacing is used to assess CTI permeability, a counterclockwise shift occurs in the collision front of the two vectors of the circumannular activation because the impulse goes back to the atria through the His Bundle and the AV node that is situated in a more superior position than the CS ostium; this means that careful mapping of the lateral side of the ablation line is essential in order to avoid missing the point of the collision front, which is shifted downwards, and therefore to avoid the mistake of regarding as blocked an isthmus that actually is not (Figure 4). Once the line of block is created, TA activation is very similar during CS pacing and RV pacing, displaying fully descending activation of the lateral wall.

### Split electrograms analysis

Analysis of double potentials on the line of block can be simplified by the use of RV pacing because retrograde atrial potentials are usually detached from the ventricular potentials and clearly visible, especially the second component. During CS pacing, the second component of the split double potentials is usually very  near  or inside the ventricular electrograms, and in some cases an extrastimulus is needed to block or prolong AV conduction and to better visualize the atrial potentials without the ventricular ones. The mean intervals separating the two components of double potentials were shorter during RV pacing than during CS pacing: as the CS ostium is nearer to the CTI than to the AV node, the first component of the double potentials is rapidly recorded by the distal pole of the ablator when positioned on the line of block, while the second component can be recorded only after activation of the entire TA has been completed. When pacing is carried out from the RV, the impulse goes back to the RA through the AV node, which is located in the septal wall above the CS ostium. Therefore, unlike the case of CS pacing, the first component of the double potentials recorded on the line of block arrives later because it has to descend the interatrial septum, while the second component arrives sooner, because it starts from the higher septum. The delay of the first component and the advance of the second one shorten the interval, as a sort of differential pacing [16-18] between a site near the CTI (i.e. the proximal CS) and a point higher up the septum (i.e. the AV node).

The shortening of the intervals between the double potentials does not interfere with their analysis. On the contrary, the delay of the first component makes it more detached from the ventricular electrograms and more visible; the advance of the second component does not interfere with the analysis of the first one, since the interval is still approximately 100 ms, which is enough to distinguish two potentials separated by an isoelectric line. The detachment from the ventricular electrogram of the second component of the double potentials during RV pacing, lets a more detailed observation of eventual changes in polarity of its initial deflection that were described as a reliable sign of conduction block  during pacing from the opposite side of the line of block [13,19] if not obscured by the overlapping of the atrial and ventricular electrograms. As already noted[8], this phenomenon is visible also in the distal electrograms of the duo-decapolar catheter whose polarity reverses at the moment of the creation of the isthmus block during RF delivery (Figure 3).  More detailed  studies are necessary to evaluate the minimum critical delay between the split double potentials that can indicate a high probability of CTI block assessed by RV pacing. In our series, the 95 % confidence level for the minimum mean value above which CTI block was completed proved to be 97 ms, which is considerably shorter than reference values used for CS pacing assessment of CTI  block[5,6].

Double electrograms are by themselves not always easy to interpret. According to previous reports, 15% of patients with fractionated electrograms can still have intact CTI conduction on activation mapping[20], due to incomplete ablation with residual or impaired conduction. In our study, all double, split and reduced-amplitude atrial potentials, separated by an isoelectrical interval, recorded during RV pacing, were associated with an evidence of circumannular activation consistent with CTI block. Despite that, our population was not randomized  and we cannot argue about the superiority of RV pacing versus CS pacing, neither of fractionated potentials on the line of block versus activation mapping.   The analysis of the first split double potential during RF delivery can be helpful in this regard. Indeed, we should look for a sudden increase in the distance between the local double potentials (a sort of 'isthmic jump', Figure 3), which indicates the moment when the CTI block is created during RF delivery, as happens when the line of block is verified by pacing from the CS [6].

### Clinical implications

Some technical applications are suggested by these observations. If retrograde VA conduction is present, pacing from the proximal CS is not strictly necessary, in that it can be substituted by RV pacing. Catheterization of the CS is often easy, quick and feasible even via the femoral route that is mostly preferred, but when anatomical variants or the presence of a redundant Tebesio valve causes precious time to be wasted, RV catheterization can be a possible rapid alternative procedure, using the same femoral vein approach. A catheter in the CS can also be avoided by properly positioning the distal poles of special catheters in the mid septal RA, but occasionally the stability of this easy set is not acceptable, because of high pacing thresholds or saturation of the atrial electrograms recorded near the pacing catheter [8] and repositioning is sometimes required. When these rare but problematic cases occur, a simple bipolar catheter in the RV is able to maintain pacing with high stability for the entire duration of the procedure.

### Limitations

The first limitation of this technique is obviously the presence of VA conduction; in the normal population, this is estimated to be about 70 % [15], while in our population it was lower (64%). If VA conduction is not present or is inadequate, we do not suggest that it should be elicited or improved by pharmacological means, such as atropine or isoproterenol infusion. At the beginning of our study, we tried these substances in patients without VA conduction, but in these cases catheterization of the CS via the subclavian or internal jugular route was more rapid than preparing the pharmacological infusion and waiting for its effect on retrograde conduction, which was not always present. Moreover, in those patients in whom these drugs induced VA conduction, administration throughout the procedure caused uncomfortable adverse effects: hypertension, tachycardia (requiring higher-frequency RV pacing), tremors and palpitations in the case of isoproterenol infusion; mouth dryness, and again tachycardia, during atropine infusion. In addition, atropine is also inadvisable in patients with prostatic hyperplasia or glaucoma. Another limitation is the possible presence of an accessory pathway that bypasses the AV node, though patients displaying this feature are usually referred for ablation of the Kent bundle rather than the CTI. As a large number of catheters were used in our study protocol, maneuvering the ablation catheter proved more difficult and procedure times and numbers of RF applications were overestimated.

## Conclusions

In patients with intact VA conduction, TA activation during CS and RV pacing is similar before and after the linear RF ablation of CTI. The occurrence of split atrial electrograms separated by an isoelectric interval registered on the line of block can be detected during CS or RV pacing. In patients with retrograde VA conduction, RV pacing is a little trick that may be helpful when CS catheterization via a femoral approach has failed or low septal atrial pacing is unsatisfactory.
